# Malaria

**Published:** 1881-09

**Authors:** 


HALL'S
### JOURNAL OF HEALTH
AND
MISCELLANY.

E. H. GIBBS, A. M., M. D. - - EDITOR.

Founded in 1854, and published Monthly without Interruption since that Time.

### MALARIA.

NO facts seemed to be better settled, until within a recent period, than that the seeds of the various diseased conditions that are generally supposed to be due to malarial poison, existed almost exclusively in marshy grounds, where water and sunshine caused the rapid decay of vegetable matter. But stubborn facts have shaken these theories that nearly all men supposed were based on the experience of ages. At present, the worst forms of malarial diseases prevail on the hills and mountains of New England, and along the Highlands of the Hudson; places that were formerly chosen as health resorts by those who were fortunate enough to get away from the unhealthy districts of New Jersey and Long Island during the heat of summer. On the other hand, I am acquainted with sections of country where until within three years hardly a family escaped fever and ague during the spring or fall months, and where during the past three years but comparatively few cases have occurred. In 1877, I published an article in the JOURNAL, in which I endeavored to prove that these diseases did not emanate from marsh lands, but could be traced more directly to organic impurities that exist in much of the water that is used for drinking and cooking. I have as yet seen no substantial reason for changing or modifying the opinion then formed.

I am acquainted with many families that have lived on the very border of extensive marshes for many years, in the enjoyment of uninterrupted health. I am acquainted with families residing in localities seemingly the most attractive in point of salubrity, being on high and dry grounds, remote from marshes or sluggish streams, but every member of which suffers more or less every year from chills and fever. I have examined the source of the water supply in many cases, and have invariably found the water charged to a dangerous degree with organic impurities. Whenever I have tested water, from wells or springs used by families who escape malarial fever, I have found it pure, or containing so little organic matter as to be hardly appreciable. In selecting a residence with reference to health I should pay less regard to the proximity of marshes and ponds than to the character of the water supply. If that is poisonous all the sanitary devices that were ever invented will not prevail against this towering evil, and sickness is inevitable. Observation has taught me to more than suspect that impure water is the principal cause of disease in places heretofore exempt.

One suggestion I wish to make in regard to fever and ague. No treatment will effect a cure unless the patient remains quiet for a few hours before and after the chill is expected. If you can once escape the regular chill it is a long stride toward recovery; and if, in addition to the proper medicine, the patient will go to bed and remain there a few hours, he will usually escape it.

